# Exostosis of the optic canal in a child – a rare diagnosis in a paediatric ear, nose and throat setting: a case report

**DOI:** 10.1186/1752-1947-8-454

**Published:** 2014-12-21

**Authors:** Milan Urík, Ivo Šlapák, Dana Pavlovská, Eva Prívarová

**Affiliations:** 8 Department of Paediatric Otorhinolaryngology, Faculty of Medicine, Masaryk University and University Hospital Brno 1, Cernopolni 9, 61300 Brno, Czech Republic; 9 Department of Paediatric Radiology, Faculty of Medicine, Masaryk University and University Hospital Brno 2, Brno, Czech Republic; 10 Paediatric Eye Clinic, Faculty of Medicine, Masaryk University and University Hospital Brno 3, Brno, Czech Republic

**Keywords:** Endonasal surgery, Exostosis, Optic canal, Optic nerve

## Abstract

**Introduction:**

Optic nerve compression is an uncommon disorder leading to deterioration or complete loss of vision.

**Case presentation:**

We describe the case of a 14-year-old Caucasian girl with a gradual deterioration of vision in her right eye. Using modern imaging techniques and endonasal endoscopic surgery, we identified the cause and removed the pathology.

**Conclusions:**

The cause of optic nerve compression was a rare exostosis in the optic canal. An endonasal endoscopic approach is the optimal choice for management of optic nerve pathologies. It is a gentle and minimally invasive surgical technique. Interdisciplinary cooperation, especially with the eye doctor and radiologist, is required in these cases.

## Introduction

The optic nerve is the first part of the visual pathway. The course of the optic nerve is divided into three segments: intraorbital, intracanalicular and intracranial [1, 2]. It begins as the optic papilla in the posterior pole of the eyeball, passes through the optic canal and continues intracranially to the optic chiasm. Compression leads primarily to visual impairment, with complete loss of vision being the most severe complication. The predisposing site for nerve compression is the optic canal, the walls of which are made of the sphenoid bone [3] providing a rigid box for the second segment of the optic nerve. Early nerve decompression has a significant effect on the extent and severity of visual impairment. Its failure leads to irreversible changes and progressive atrophy of the optic nerve. The most common cause of compression is injury in the craniofacial region; less common causes include inflammation of the nerve, a tumour infiltrating from the sella turcica area or thyroid-associated orbitopathy [4–6]. Exostosis in the optic canal is a rare pathology leading to optic nerve compression.

## Case presentation

**Figure 1:**
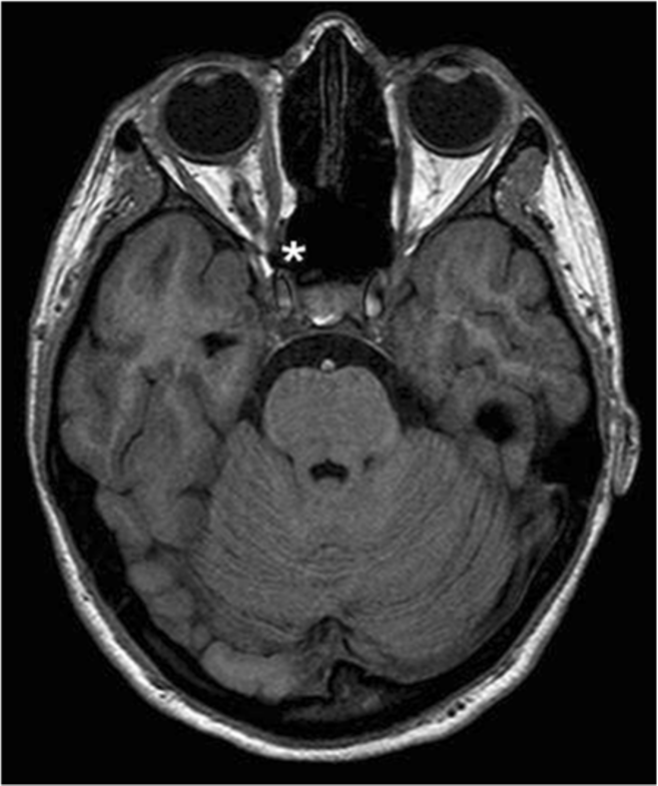
Pathology of the right optic nerve (*) – postcontrast magnetic resonance imaging.

**Figure 2:**
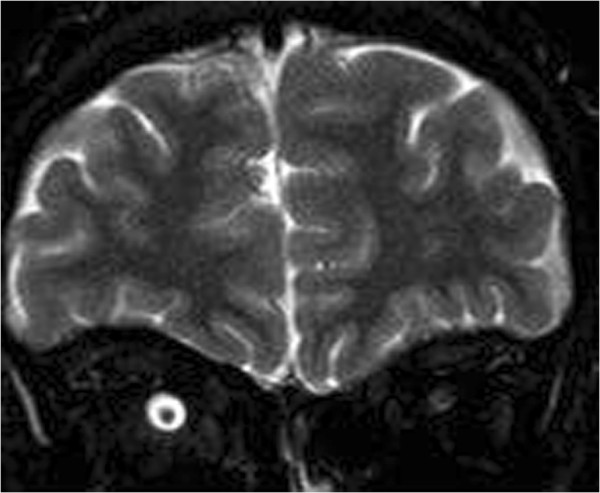
**Compression of the optic nerve, right side, magnetic resonance imaging.**

**Figure 3:**
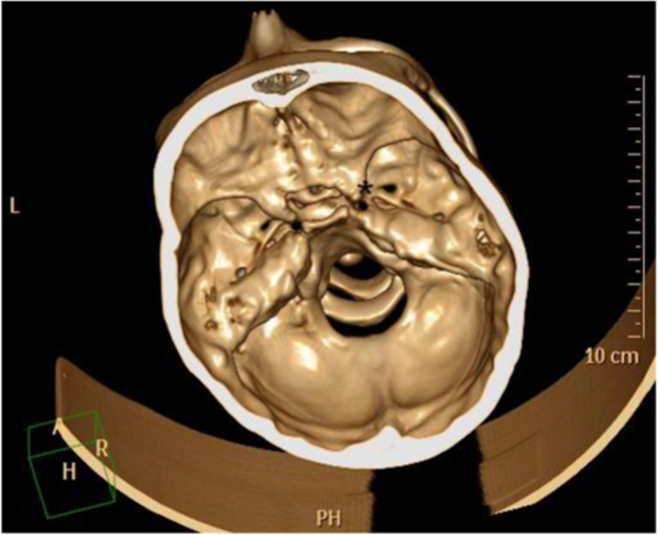
Robust processus clinoideus (*) on the right side – three-dimensional computed tomography reconstruction.

**Figure 4:**
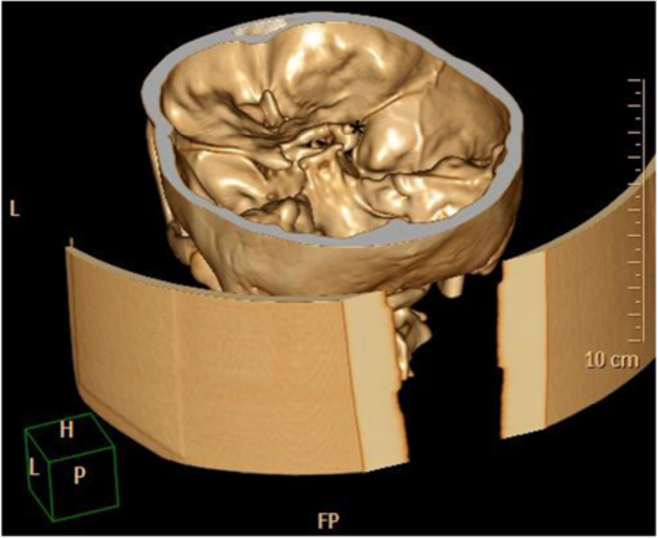
Robust processus clinoideus (*) on the right side – three-dimensional computed tomography reconstruction.

**Figure 5:**
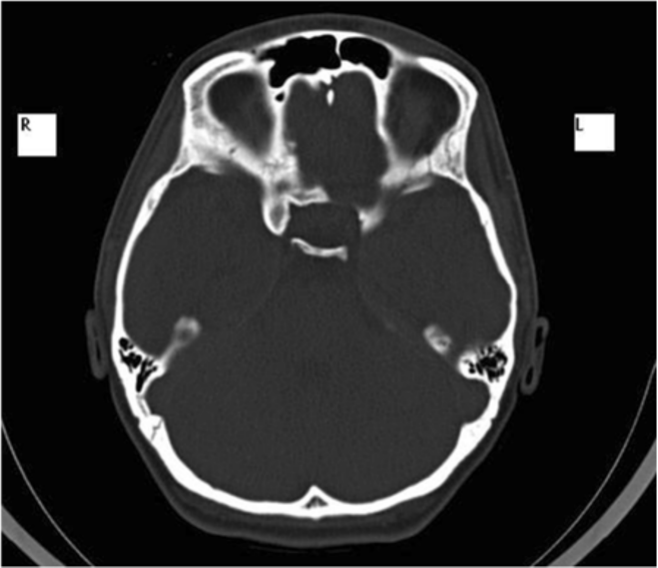
**High-resolution computed tomography scan, axial projection.**

**Figure 6:**
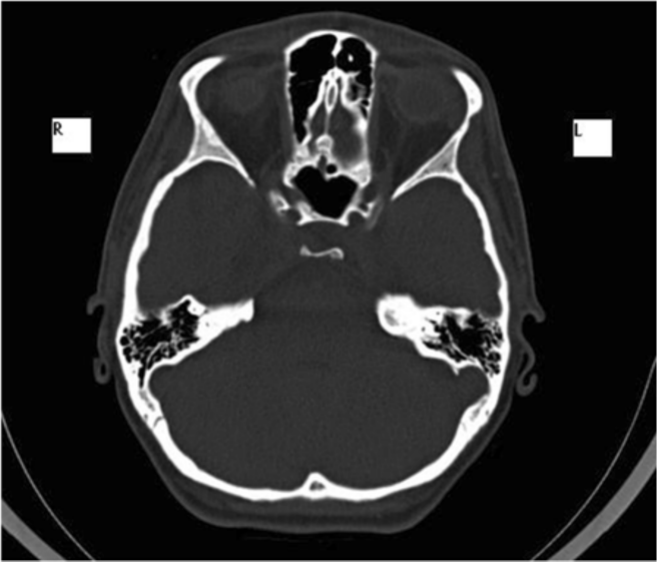
**High-resolution computed tomography scan, axial projection.**

In February 2007, a 14-year-old Caucasian girl was admitted to our Ear, Nose and Throat department. Since the beginning of 2006 she has been monitored for gradual loss of vision in her right eye. At the out-patient eye clinic of our hospital, she was diagnosed with reduced vision in her right eye due to partial atrophy of her optic nerve. Her vision ranges from 2 to 4m; the use of optical correction fails to improve the condition. The position of her eyes is parallel, her eyeballs are movable in all directions, the anterior segment is intact, optic media are transparent, and pale atrophic papillae are seen in the right fundus; the findings are unchanged without progression. The findings in the left fundus are physiological. Baseline perimetry revealed absolute central scotoma up to 25 degrees, which coincides with the blind spot. Eye findings correspond to optic nerve compression. In May 2006, magnetic resonance imaging (MRI) scanning was performed with the conclusion of suspected tumour of the prechiasmatic segment of her right optic nerve. In the summer of 2006, a temporary improvement in clinical symptoms occurred; however, during the autumn of the same year her symptoms worsened again and she developed headaches in addition to visual problems. In December 2006, a follow-up MRI scan was performed and revealed significant enlargement of the subarachnoid space of her right optic nerve (Figures 1 and 2). An enlarged bony structure was also seen laterally in the optic canal area, which was surrounded by solid tissue, probably compressing the optic nerve in the optic canal. An additional computed tomography (CT) scan was performed, which revealed a robust anterior clinoid process (Figures 3, 4, 5 and 6) and otherwise no further pathology.

**Figure 7:**
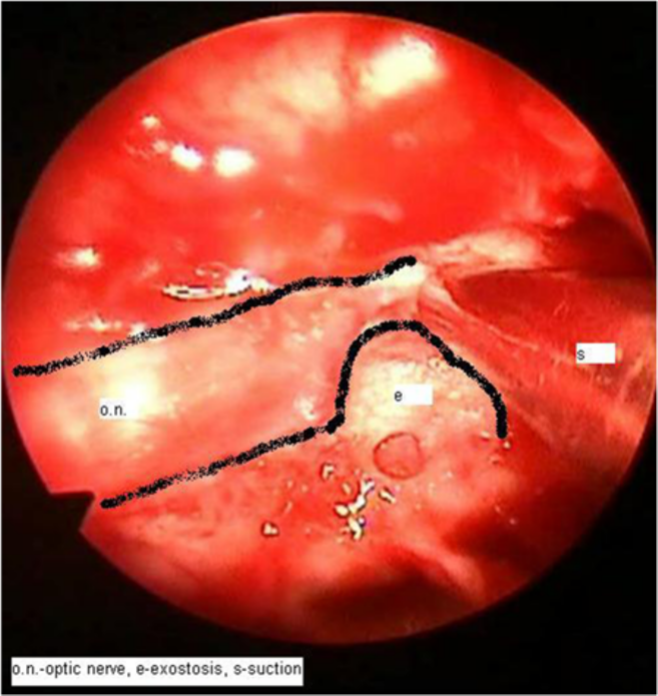
**Endoscopic endonasal surgery – exostosis and compression of the optic nerve.** Abbreviations: e, exostosis; o.n., optic nerve; s, suction.

**Figure 8:**
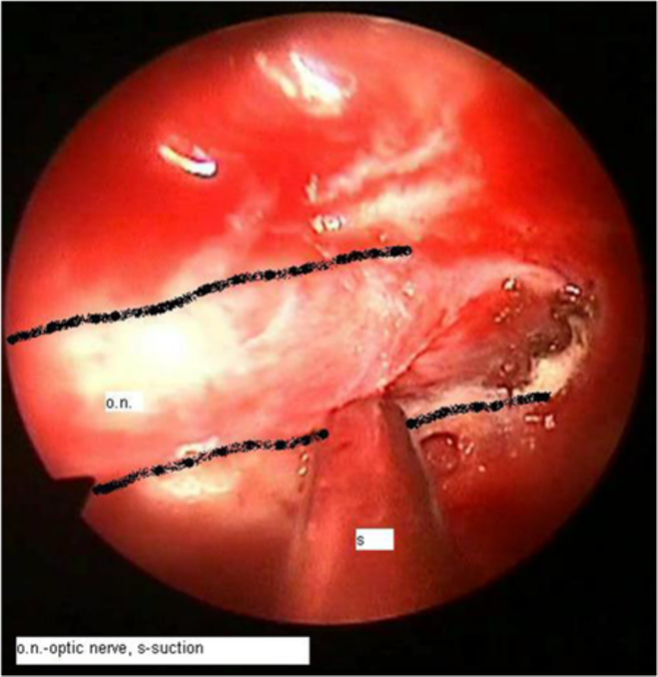
**Endoscopic endonasal surgery – the view after eradication of exostosis.** Abbreviations: o.n., optic nerve; s, suction.

Endonasal endoscopic decompression of her optic nerve was performed (Figures 7 and 8). Medial and posterior ethmoidectomy and sphenoidotomy were performed under general anaesthesia using endoscopic techniques; subsequently bone was removed from the medial wall of her orbit and her optic canal was gradually approached. The optic canal narrowing was seen about 13mm behind the tip of her orbit, which enlarged again in the direction towards the chiasm. A part of the bone in the narrowed area was removed using punches, shaver and diamond bur to release the optic nerve. The histology report described the bone formation as a lamellar compact bone. She was subsequently referred to the care of an ophthalmologist and underwent regular eye examinations. Her condition markedly improved, with persistent central scotoma in her right eye.

In May 2008, her vision worsened again in her right eye, and she complained of blurred vision without diplopia. A CT scan revealed narrowing of the right optic canal and more robust optic nerve in her right eye, without other obvious pathology in the canal or sella turcica. An eye examination revealed reduced vision in her right eye, with non-progressing atrophy of her optic nerve; otherwise the findings were unchanged compared to the previous evaluations. She was admitted for revision endonasal endoscopic surgery, during which narrowing of her right optic canal was revealed due to bone apposition similar to bone exostosis. This bony process was removed using a cutter and her optic nerve was released. After surgery, her visual acuity gradually improved.

In January 2010, she presented for recurrent visual impairment in her right eye, without obvious pathology according to the CT scan. Revision endonasal endoscopic surgery was performed, during which her right optic nerve was not compressed over its course in the optic canal, but was more robust than her left optic nerve. New pathology was ruled out and an MRI scan was recommended, which confirmed a robust right optic nerve compared to the thickness of her left optic nerve. It is probable that the thickening of the nerve developed as a result of pathological processes that took place. The thickening of the nerve has a stationary character. Currently, he is stabilized and followed up by her ophthalmologist. She achieved a significant improvement in visual acuity, which is stable at 4m, follow-up perimetry in 2014 revealed a persistent central scotoma in her right eye, including partial scotoma in the upper temporal quadrant. Perimetry of her left eye provided physiological findings, without any failures in her visual field.

## Discussion

We should always consider the possibility of optic nerve compression in patients with progressive loss of vision, particularly in those with unilateral symptoms. The definitive diagnosis of optic nerve compression is determined based on the results of imaging techniques, in particular MRI or CT scanning. Our case demonstrated that modern imaging techniques are crucial and able to detect even a tiny bone abnormality that caused severe clinical problems.

Naturally, treatment of compression depends on the cause of the disease. Some authors believe that administration of high doses of corticosteroids is superior to surgical treatment [7, 8], whereas others advocate surgical decompression in patients with clear evidence of optic nerve compression due to bone fracture [9, 10]. Prioritising surgery over administration of high doses of corticosteroids in patients with traumatic optic neuropathy is still a controversial issue [1, 9]. The aim of decompression surgery is to eliminate the causes of compression while minimizing the oedema leading to secondary nerve damage.

Timely surgical intervention is required in patients with compression caused by mechanical compression of the nerve by another structure, such as a tumour, haematoma or, in our case, bone exostosis. Several surgical approaches are used to achieve adequate decompression of the optic nerve, including transfacial, transcranial or endoscopic endonasal approaches [9]. The optimal surgical approach should be chosen according to the location of the compression. The transcranial approach is preferable if the decompression is located in the vault of the optic canal, whereas the endonasal endoscopic approach is suitable for inferomedial access to the optic canal [11].

An endonasal endoscopic transsphenoidal approach has become increasingly preferred over conventional microsurgical techniques, as it is a gentle and minimally invasive technique [12, 13], which enables elegant removal of the causes of compression and shortens the length of hospitalization and recovery of the patient. The endoscopic endonasal technique has several advantages such as lower morbidity, better protection of the optic nerve, rapid patient recovery time, more acceptable cosmetic effect without scars on the face and head, and no risk of damage to the teeth in children [1].

## Conclusions

Although developing over a long period of time, optic nerve compression is a serious condition that can lead to complete loss of vision. Therefore it requires an efficient treatment that should be as fast as possible. This case study describes a relatively rare case of optic nerve compression caused by bone structures developed from pathological ossification of the optic canal. At the same time, we wanted to emphasize the possibility of using very gentle and minimally invasive surgical techniques, such as an endonasal transsphenoidal approach that can be used for optic nerve decompression. Interdisciplinary cooperation, especially with the eye doctor and radiologist, is required in these cases.

## Consent

Written informed consent was obtained from the patient's parent for publication of this case report and accompanying images. A copy of the written consent is available for review by the Editor-in-Chief of this journal.

## Authors’ information

Milan Urík, MD is a doctor and an assistant of Department of Paediatric Otorhinolaryngology. He is interested in otology and endonasal surgery.

Ivo Šlapák, MD, Professor is the head of Department of Paediatric Otorhinolaryngology and the best specialist for endonasal surgery in childhood in the Czech Republic.

## Abbreviations

CT: Computed tomography
MRI: Magnetic resonance imaging.
